# On Hydrophobia

**Published:** 1808-11

**Authors:** 


### *To the Editors of the Medical and Physical Journal.*

Gentlemen,

THERE is nothing more destructive of true science, than acquiesence in philosophical conclusions, without reiterated and cool examination; and there is probably, no science, in which there is more of hasty assumption, without proof, than in that of medicine. To the inexperienced and sanguine, every thing is clear and certain. A patient, employing a remedy new to the prescriber, recovers; immediately the means are announced as specific and infallible. Another is bled in a pleurisy, and dies. The practitioner incontinently inveighs against blood-letting as a vile practice, which ought to be banished from all learned and even civilized society. A third proposes for your immediate solution, questions, which involve half the topics of pathological knowledge; and a fourth pronounces on the principles of medical science, as if it were as easy to fabricate a system, as to make a shoe.

Now it might, at first view, appear of little moment, that men should thus exhibit themselves before the public. What matters it that the farces are damned, and the authors forgotten? There is, however, this evil, — that no folly is without its patron, with talents precisely commensurate to their object; and medical absurdities are never wholly relinquished without much expence of human ease and human life.

These reflections have often suggested themselves to me, in perusing your Journal; but seldom more forcibly than from various articles in that Number which I have this day received, and more especially from a Dissertation on the Functions of the Liver. This dissertation abounds with queries, to answer which, requires from the author some indirect and positive proofs of the doctrines which he insinuates, in language not sufficiently precise for physiology.

The subject of hydrophobia has of late much and justly occupied the public attention; it is therefore indispensable to relate cases and to propose cures of hydrophobia. One gentleman, in the Number of your Journal before me, describes four animals, which were delirious, and howled and bit, and of which one refused food. But are these sufficient proofs that they were affected with hydrophobia, or dread of swallowing liquids?

Let us examine other points. On the 21st of June, the dog bit master Hill. We are told that he was a fierce creature, whom the child had been accustomed to provoke, and at whom he had snapped once or twice before. At the time in question, he was engaged in a pursuit, in which few animals, whether brute or human, suffer interruption without peevishness. He was following a *proud bitch*; when master Hill coming in his way, all his recollections came at once across across him, and he seized the boy by the heel. But was he hydrophobous? No. "He had never refused food, nor exhibited any symptom of indisposition." Now, the natural business in which the dog was engaged, together with the want of symptoms, where, if they had existed, they must easily have been perceived, afford a high probability that, at this time, he was actually in health; and this probability will not be invalidated by his having been daily in the streets, or by the general opinion, that there were several mad dogs in the neighbourhood, unless it can be proved that these dogs in the neighbourhood were actually mad; and, if they were mad, that they had infected the dog in question.

Two days afterwards, on June 23, the same dog bit a maid-servant, a bitch, and two cats. On the 25th, he alternately howled, and bit, and tore; and now four days had elapsed since he had bitten master Hill, when, unfortunately for the investigation of truth, he was killed. It is, however, of considerable consequence to attend to this interval, because I doubt whether any dog ever lived four days after having begun to be affected with symptoms of the true rabies canina. But to proceed.

On the 2d of July, one of the cats bitten on the 23d of June, seized a girl a little above the ancle, and bit her in four places. The cat was killed, and thus, a second time, the course of the malady was interrupted, and the event concealed. Was this cat hydrophobous? If it was, the progress of disease was somewhat quick: for it was fully established in nine days. This circumstance is strongly against the conclusion, that the disease was the rabies hydrophobica, the period at which that malady appears being rarely so short a time as three weeks, and being more usually six weeks after the bite: besides which, after the first appearance of the symptoms, at least one day commonly elapses before the animal falls upon and bites others. The bitch also sickened about the same time, though with no symptoms of hydrophobia, and died on the 7th of July; and this coincidence of deviation from the common progress of infection, affords, in my mind, a still stronger presumption that the disease was of a different kind from that which the author apprehended.

With regard to treatment, nothing can be more worthy of praise than that which he employed. But the inference is not so certain, viz. "that the fact of preservation is evidently made out." Many a person recovers under the use of remedies, which have no share in effecting the recove-

 ry.

ry. The nurse, who watches the patient in typhus, is not preserved from infection by the camphor bag which she wears on her breast; and on this very subject, if the author will only consult the Treatise of the late Dr. James, he will find, among other valuable information, that which I will spare him the trouble of seeking. "I once knew a footman," says this sagacious physician, "belonging to a very near relation of mine, who was three times bit by dogs manifestly mad, insomuch, that several animals bit by the same dog, near the same hour, contracted the distemper and died. This fellow was so obstinate, that he could not be prevailed upon to do the least thing by way of precaution; and yet never had the least tendency to an hydrophobia; so far from it, that he died many years after, in consequence of drinking too much, at an ale-house in Whitechapel, of which he was master." Treatise on Canine Madness, p. 40.

A case of hydrophobia is related by another author. A young man, in a salivation from mercury, had, on the 31st of July, an inflammation of the fauces and uvula, and complained that he had some difficulty of swallowing. Notwithstanding which, he eat his dinners with good appetite, except so far as it was lessened by a depression of spirits. Hitherto, I should presume that there is no evidence of hydrophobia. In the afternoon, however, he had a tickling in his throat; and, after having fainted, he remained for five minutes in a comatose state, from which he was suddenly roused by cold water forcibly thrown in his face. Soon afterwards, the poor young man became vehemently convulsed, suffering globus hystericus, foaming at mouth, and affected with stertorous respiration common in disorders of the epileptic type. The author indeed observes, that "this last symptom is universally attendant on hydrophobia." Should he not have added, "in its very last period?" when, as at the conclusion of various other fatal diseases, a difficulty of respiration may for a short time before dissolution, seem to exceed the diminution of the other vital powers. The patient, after having been thus exhausted by struggles, in which three men were scarcely able to confine him, fell down into a state of coma, from which the author roused him as before by affusion of cold water, only to witness a repetition of similar sufferings.

Such might be the nature and course of hysterical symptoms, approaching to epilepsy, which the author, with a proper diffidence, expressed his suspicions might be hydrobia; for the word operated as a stimulus equally effectual with with the cold water. It roused the patient from his trance, he laughed sardonically, and raved, though without fear, about having been bitten by a dog. A fit of tears however supervened, and with a little subsequent sleep, happily carried off the symptoms, which began and ended within twelve hours.

Whether the patient in this case was unwilling to drink we are not told; but we learn, that after his sleep he drank with great ease and avidity; and, probably, he might feel indignant that a glass of liquor should have been thrown over him.

Let us now attend to the dog Ponto, respecting whom it is of importance to make three inquiries;

1st. Whether Ponto really existed excepting in the patient's imagination?

2dly. Whether the patient was bitten by Ponto? and,

3dly. Whether Ponto was mad?

I turn from this case to one of a graver nature, by an author, whose very errors are respectable. Master Todd, aged 5 years, bitten on the 6th of August by a mad dog in the cheek near the mouth, was seized with a fever on the 9th of September, beginning as the usual remittent, but unsuccessfully treated by bleeding, purging, blisters, and the warm bath, though "the last named remedy appeared to afford him some relief, which he manifested by playing and paddling in the water." "He discovered no dread of water, except in one instance, when he turned from it with horror. He swallowed occasionally about a spoonful of it at a time. Immediately after, he looked pale, and panted for breath. He spoke rapidly and with much difficulty. After speaking, he panted for breath in the same manner that he did after drinking. He coughed and breathed as patients do in the moderate grade of the cynanche trachealis." These symptoms appear to have taken place on the 13th of September, the doctor having seen him only six hours before his death, which happened suddenly, about twelve o'clock in the night of that day.

Let us pause to consider these symptoms. I would ask any experienced medical practitioner, whether they at all resemble what occurs in the true canine hydrophobia; and whether, on the contrary, they are not precisely such as occur in cases of inflammation about the pharynx and larynx? In such cases, speaking and swallowing must unavoidably produce great pain, and at the same time difficulty of respiration. But are difficulty of swallowing, and consequent dread of drinking, of themselves, sufficient to

 characterize characterize hydrophobia? Do we not meet with them in scarlatina? in which the patient dreads the consequences of drinking to such a degree as never to drink at all. In the true hydrophobia canina, not only the act of swallowing, but the touch, the sight, and at length even the name of liquid, produces a kind of convulsive horror. This patient, on the contrary, not only received pleasure from paddling in water, but occasionally swallowed a spoonful of it at a time. His breathing resembled that of patients in the croup; and he died suddenly, probably from a spasmodic stoppage of respiration, as takes place usually in the former disease. His death happened on the 5th day from the commencement of the symptoms; which is from thirty-six to forty-eight hours longer than any one was ever known to live under unambiguous symptoms of the canine hydrophobia.

The having been bitten by a dog affords, as I have before observed, of itself, no presumption of the nature of the disease,* unless the dog was proved to be mad. That that was the case in the instance before us, there is no attempt to shew. And though in such cases, madness were the usual consequence of a bite, it would not follow that every person dying a month after the bite of a rabid animal, died *from* that bite. In order to ascertain the reality of the disease, the symptoms must correspond with those which are usually found to follow similar accidents. Every object must have its *genus proximum* and *differentia;* that is, invariable qualities, not only in which it agrees, but in which it differs from every other object or genus. If, regardless of such a distinction, we consider phænomena merely according to their succession, a man who after having been bitten by a dog happens to be visited by a corn on his toe, may be said to be hydrophobous.

I have shewn the want of these distinctive qualities in the disease before us, which, in reality, the dissection itself proved to be a vehement inflammation in the trachea and other parts adjacent.

If, however, the disease had been actually identified, were the proposed remedy likely to relieve it? Inflamma-

* On account of the importance of the subject, we have admitted these anonymous remarks on authors who have honoured us with their names; yet, in defence of the latter, and for the better information of C. B., we must remark, that Hydrophobia is no necessary symptom of rabies in dogs. We shall be thankful for the further Communications of this ingenious writer; and if he adds his name, shall take fewer liberties with them.

tion tion of the throat does not necessarily exist in rabies canina. I have seen a case of that kind, which proved fatal in about sixty-five hours, in which the most accurate dissection shewed no vestige of disease whatever in any part of the membranes, substance or cavities of the brain, fauces, trachea, œsophagus, stomach, intestines, or any other of the thoracic or abdominal vicera. The same thing happens in the tetanus or trismus, in which, in several instances, I have observed the same exemption from all morbid appearances. If the patient no more dies of the affection of the throat in hydrophobia, than of the locked jaw in trismus, precisely the same benefit may be expected from laryngotomy in the former disease, as from the vulgar remedy of wrenching open the jaws or drawing the teeth in the latter. In both cases the constitution may be labouring under desperate and hitherto remediless maladies, of which the difficulty of swallowing and the rigidity of the jaws, may be only local, and, if I may be allowed the expression, unimportant symptoms.

Medical practitioners should suspect their own observations in rabies canina, Non cuivis contingit adire Corinthum. The real malady is so rare, that he would probably be speaking much within bounds who should assert, that a man may have attended 50,000 patients, without having, in his own practice, seen a single case of that formidable diseae.

Should these strictures be acceptable, I may possibly, on some future occassion, again address you; in the mean while I am, &c.

